# Eco-Friendly Ca-Montmorillonite Grafted by Non-Acidic Ionic Liquid Used as A Solid Acid Catalyst in Cellulose Hydrolysis to Reducing Sugars

**DOI:** 10.3390/molecules24091832

**Published:** 2019-05-13

**Authors:** Yang Zhou, Miao Yang, Dongshen Tong, Haiyan Yang, Kai Fang

**Affiliations:** State Key Laboratory Breeding Base of Green Chemistry Synthesis Technology, Discipline of Industrial Catalysis, College of Chemical Engineering, Zhejiang University of Technology, Hangzhou 310032, China; zy15958048374@163.com (Y.Z.); 2111601217@zjut.edu.cn (M.Y.); 2111701038@zjut.edu.cn (H.Y.); 2111701376@zjut.edu.cn (K.F.)

**Keywords:** montmorillonite, ionic liquid, cellulose, hydrolysis, reducing sugars

## Abstract

An effective and friendly method was developed for the production of reducing sugars (RS) from the hydrolysis of cellulose over the solid catalyst of Ca-montmorillonite (Mt) grafted by non-acidic ionic liquid (Mt-IL) in water. The effect of mass ratio, water dosage, reaction temperature, and time were investigated in a batch reactor. Raw Mt showed only a 7.9% total reducing sugars (TRS) yield for the catalytic hydrolysis of cellulose in water. As the Mt was grafted by -SO_3_H and IL, the TRS yield greatly increased under the same reaction conditions. The highest TRS yield of 35.7% was obtained on the catalyst of Mt grafted by non-acidic IL at 200 °C with the mass ratio of catalyst to cellulose of 0.2 for 120 min. The high TRS yield for Mt-IL should be attributed to the synergistic effect of the dissolution of cellulose by IL and the exposed metal ions on the layer with water. Although the yield of TRS on Mt-IL decreased gradually with recycling runs, the decrease after the first run was not very serious compared to the fresh catalyst. This work provides a promising strategy for efficient cellulose hydrolysis into fine chemicals by Mt with non-acidic IL.

## 1. Introduction

The depletion of fossil fuel resources and global climate change have led to more and more attention on developing a series of novel chemicals based on renewable feedstocks. Many studies have been devoted to converting renewable biomass to useful chemicals and clean fuels [1,2,3]. Among the natural biomass, cellulosic biomass is the most abundant inedible biomass resource produced by photosynthesis from CO_2_ and H_2_O. Recently, the conversion of cellulose to value-added chemicals has been extensively studied [4,5,6]. Since reducing sugars (RS)—mainly glucose—are platform molecules for production of other chemicals, such as 5-hydroxymethylfurfural (HMF), levulinic acid, and alkylglucosides, depolymerization of cellulose to RS plays a critical role [7,8].

Cellulose is a water-insoluble aggregate of long-chain β-1,4 glucan composed of glucose monomers linked by β-1,4 glycosidic bonds. Hydrolysis of β-1,4 glycosidic bonds and decomposition of hydrogen bonds linking β-1,4 glucan chains into water-soluble saccharides are the main technology to obtain RS [9,10,11,12]. Up to now, several technologies in the hydrolysis of cellulose have been applied, such as enzyme hydrolysis, mineral acid hydrolysis, and supercritical water hydrolysis [13,14,15]. Generally, enzymatic hydrolysis is the most promising approach due to its low temperature and high activity, but the high cost and reuse of the enzyme inhibit its commercialization. Mineral acids are effective for cellulose hydrolysis, however, the catalyst recovery and the waste water treatment are the problems. Supercritical water hydrolysis is also an efficient process, but it has drawbacks, such as harsh conditions and many side-reactions [16,17,18]. On the contrary, due to the advantages of easy separation and recycling, hydrolysis of cellulose over heterogeneous catalysts has received greater attention in recent years [19].

Several groups have reported that cellulose was hydrolyzed in the presence of solid acid catalysts, such as Amberlyst 15 [20], heteropolyacids [21,22], magnetic solid acid catalyst [23,24], and functional carbon materials [25,26]. Among them, porous solid materials modified by the -SO_3_H groups are extensively investigated for their cheap and easy preparation [27,28]. Although the catalysts supported by -SO_3_H groups show strong acidity and high activity in the hydrolysis of cellulose, -SO_3_H groups are easily leaching out and lead to environmental problems. Moreover, the hydrolysis efficiency is also low due to mass transfer resistance between solid acids and insoluble cellulose in water [29]. Consequently, more effort should be devoted to developing the environmentally friendly and efficient solid acid catalysts for the hydrolysis of cellulose.

Clay minerals are a class of phyllosilicates, which are ubiquitous in nature [30]. Depending on their layer structure, high specific surface area, and ion exchange capacity, a great number of new clay-based materials have been developed with fascinating functionalities [31,32,33]. Among clay minerals, montmorillonite (Mt) is one of the most common 2:1 type cationic clay with highly hydrothermal stability and thermal stability [34,35]. Functional Mt has received much attention over the past decades because it is useful in many fields, including adsorption [36], catalysis [37], and separation [38]. In our previous work, acid-activated Mt had been studied for the hydrolysis of cellulose in water, which showed higher activity than that of ZSM-5, but the total reducing sugars (TRS) yield was low [39]. In the past decade, due to the interaction between Cl^−^ of the ionic liquid (IL) and hydroxyl groups of the polysaccharides [40], IL had been widely used for the dissolution of cellulose under mild conditions [41,42]. Moreover, the acidic functionalization of IL can directly catalyze the hydrolysis of cellulose with the ability of dissolution. Recently, more and more attention has been focused on the hydrolysis of cellulose in the IL [43,44,45]. However, there are still some problems, such as the high cost, high viscosity, and difficulty in separation of IL [46,47,48]. Therefore, from the view point of green chemistry, more efficient ways should be developed for the utilization of IL. 

In this work, a new heterogeneous catalyst was prepared by grafting the 1-(trimethoxy propyl silane)-3-methyl imidazolium chloride groups without acidic groups on the surface of Mt (Mt-IL). The catalytic activity and reuse of Mt-IL were examined by the hydrolysis of cellulose into RS in water. The process provided a green and efficient method for the efficient hydrolysis of cellulose catalyzed by the weakly acidic material grafted by non-acidic IL.

## 2. Results and Discussion

### 2.1. Characterization of Catalysts

**Figure 1:**
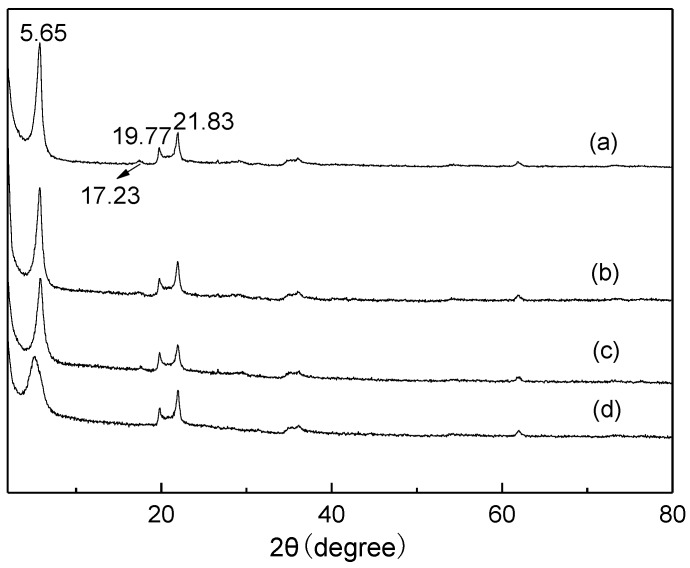
XRD patterns of Mt (**a**), HMt (**b**), Mt-SO_3_H (**c**), and Mt-IL (**d**). IL: ionic liquid.

The X-ray diffraction (XRD) patterns of natural and modified montmorillonites are shown in Figure 1. From Figure 1, the XRD pattern of raw Mt clearly displayed (001) diffraction peak at about 5.65°, indicating the basal spacing is 1.56 nm as calculated by Bragg’s equation, and agrees with that of Ca-montmorillonite (Ca-Mt). The diffraction peaks at 17.23° and 19.77° were corresponding to the (003) and (020) planes, respectively. The diffraction peak at 21.83° was related to the impurities, such as quartz and calcite [49,50]. For HMt, the diffraction peaks were almost similar with that of raw Mt, presenting that the layer structure was not destroyed after the acid treatment [51]. As the Mt was modified by -SO_3_H groups, the diffraction intensity of the (001) plane weakened, presenting that the crystallinity of Mt decreased after the modification of -SO_3_H groups on the surface. For Mt-IL, it was obvious that the (001) diffraction peak was shifted to the lower angle, moreover, the diffraction intensity weakened, and the width also became a broad comparison with the raw Mt. This result presented that the interlayer of Mt was intercalated by IL groups and the crystallinity of Mt decreased a lot. 

**Figure 2:**
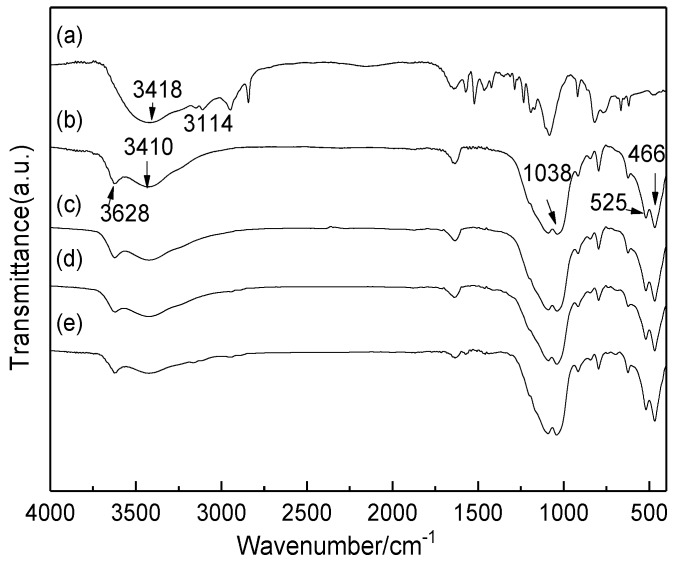
FT-IR spectra of IL (**a**), Mt (**b**), HMt (**c**), Mt-SO_3_H (**d**), and Mt-IL (**e**).

Figure 2 shows the Fourier transform infrared (FT-IR) spectra of IL, raw Mt, and the modified Mt. According to Figure 2, the two adsorption peaks for IL at about 3418 and 3114 cm^−1^ were ascribed to the aromatic N-H and C-H, respectively. Meanwhile, it also showed the typical bands of the imidazole ring at 1600–1400 cm^−1^ [52]. For raw Mt, two absorption peaks around 3410 and 3628 cm^−1^ were ascribed to the stretching vibration of the OH groups. The band at about 1038 cm^−1^ was due to the stretching vibration of the Si-O. The bands at 525 and 466 cm^−1^ were attributed to the stretching and bending vibrations of Si-O-Al, respectively [51]. The FT-IR spectra of HMt and Mt-SO_3_H showed the similar vibration bands with that of Mt. It presented that Al cations in the octahedral layer of Mt were not leached out after the acid treatment and the structure was not destroyed. In addition, because there existed the overlap of the characteristic bands between -SO_3_H and Mt, the S=O vibration bands at 1140 and 1034 cm^−1^ were not observed from Mt-SO_3_H [53]. For Mt-IL, due to the overlap of the characteristic bands between IL and Mt, the characteristic bands belonging to the IL groups were also hardly observed, but it still showed the characteristic vibrations for the polycyclic aromatics in the 1400–1600 cm^−1^ region. It also presented that the -SO_3_H and IL groups were grafted on the surface of Mt.

**Figure 3:**
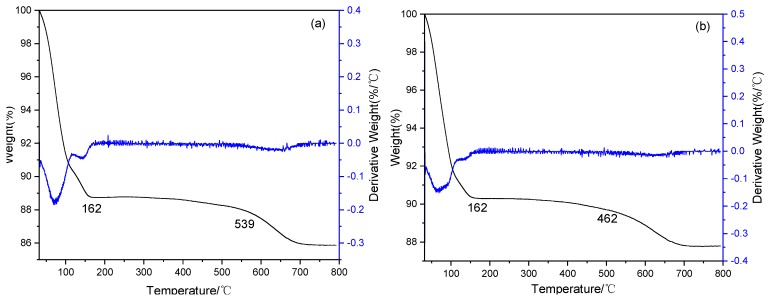

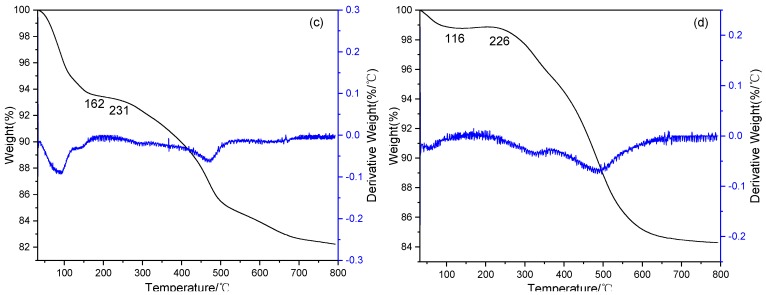
Thermogravimetric-differential thermogravimetric (TG-DTG) curves of Mt (**a**), HMt (**b**), Mt-SO_3_H (**c**), and Mt-IL(**d**).

The thermal stability of the materials described above was checked by the thermogravimetric analysis (TGA) technique and their thermogravimetric (TG) and differential thermogravimetric (DTG) curves are shown in Figure 3. These materials generally showed three main peaks for loss of weight in the range 30–800 °C. For Mt, three major weight losses were observed. The maximum weight loss peaks at 70 °C were mainly due to the volatilization of surface adsorbed water. The weight loss around 140 °C was attributed to the dehydration of interlayer water and hydrated cations on the Mt, while the maximum weight loss peak at 640 °C was ascribed to the dehydroxylation processes [54]. When -SO_3_H and IL groups were introduced into the Mt, two new peaks around 295 and 325 °C were observed on the DTG curves of Mt-SO_3_H and Mt-IL, which should be contributed to the decomposition of the surface’s -SO_3_H and IL groups anchored on the surface of Mt. The fourth consecutive peak appearing between 400 and 700 °C might originate from the decomposition of siloxane groups grafted on the surface of Mt and the dehydroxylation on the skeleton of Mt. Obviously, in comparison with the DTG curve of Mt-SO_3_H, the maximum weight loss peak of the Mt-IL was shifted to the higher temperature in the range 400–700 °C. This might be that there existed the intramolecular hydrogen bond interaction between IL and the surface Si-OH of Mt, which should be favorable to improving the thermal stability.

### 2.2. Hydrolysis of Cellulose

#### 2.2.1. Effect of Mt Modified by Different Functional Groups in Cellulose Hydrolysis

Table 1 shows the hydrolysis of cellulose in water catalyzed by the Mt and functional Mt. As a reference reaction, cellulose hydrolysis in water under the Mt catalyst had a TRS yield of 7.9% with the 5-HMF yield of 0.4%, which should be attributed to the role of H^+^ from the surface of Mt. It is well known that acidity is the important factor affecting the performance of the catalysts for the cellulose hydrolysis. It was obvious that the TRS yield was up to 14.4% under the acid activated Mt (entry 2), which should be attributed to the increase of the acidity on the catalyst. It was also interesting that although the acidic sites of HMt were much more than that of Mt-SO_3_H (entry 3 and 4), Mt-SO_3_H showed the higher TRS yield than HMt. It might be that the H^+^ ions of HMt were mainly in the interlayer of Mt and the large cellulose molecules difficultly entered into the layer of Mt, which led to the TRS yield decline. With the increase of the -SO_3_H amount on Mt-SO_3_H samples (entry 3, 4, and 5), the TRS yield firstly increased and then changed little, and finally the highest TRS yield of 24.6% was obtained. This phenomenon could be explained by the high acidity of Mt-SO_3_H associated with the ineffective dissolution of cellulose, which results in a slower hydrolysis of cellulose into RS and then rapid degradation of cellulose to other byproducts. According to Table 1, it was fascinating that the Mt modified by non-acidic IL showed the highest TRS yield with the highest HMF yield for the hydrolysis of cellulose under water in the tested samples. Practically, negligible hydrolysis was observed for non-acidic IL, such as [BMIM][OTF] or [BMIM][Cl] [55]. It had been reported that for clay minerals, the exposed metal ions (M^n+^) on the ending of the layer could interact with water and formed the structure of M^n+^-OH^−^-H^+^ [56]. Therefore, in this study, the H^+^ ions were released from M^n+^-OH^−^-H^+^ in water, and it could be reasonably deduced that the high TRS yield for Mt-IL should be attributed to the synergistic effect of the dissolution of cellulose by IL and the exposed metal ions on the layer with water. Mt is a bio-compatible and eco-friendly material with high hydrothermal stability and thermal stability. Moreover, it is ubiquitous in nature. Thus, the novel catalyst is a promising and environmentally friendly material for cellulose hydrolysis. 

#### 2.2.2. Effect of Reaction Conditions on Cellulose Hydrolysis over Mt-IL

**Figure 4:**
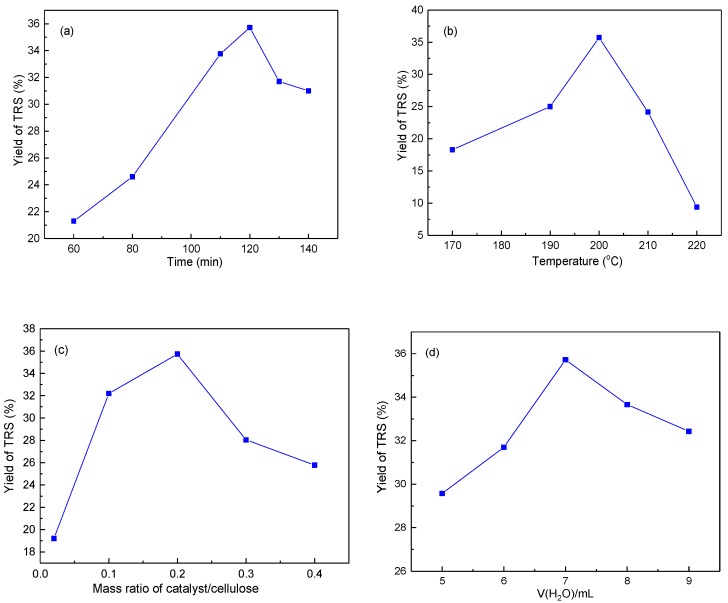
Effect of reaction time (**a**), temperature (**b**), weight ratio of catalysts to cellulose (**c**), and water dosage (**d**) on TRS yield over Mt-IL.

The effect of reaction conditions, such as reaction time, temperature, and the ratio of catalyst to cellulose on the catalytic performance of Mt-IL, were investigated in order to maximize the yield of TRS. According to the reports, RS could be further transformed to other products with the increasing of reaction time, such as levulinic acid, formic acid, or coke [57,58]. Thus, the effect of reaction time for cellulose hydrolysis was firstly investigated at 200 °C and the results are shown in Figure 4a. According to Figure 4a, the yield of TRS increased obviously at the initial stage. After reaching the maximum yield of 35.7% in 120 min, the TRS yield slowly reduced from 2.5 to 3.5 h, which might be the conversion of the formed RS. Figure 4b presents the effect of reaction temperature on TRS yield. The yield of TRS increased from 18.3% to 35.7% as the temperature was elevated from 170 to 200 °C. Subsequently, the TRS yield decreased with the increasing temperature. This might be that the RS decomposed to other products or formed the coke on the surface at high temperature [39,59]. Figure 4c shows the effect of the weight ratio of catalyst to cellulose on the TRS yield. The TRS yield firstly increased and then decreased with the increasing of the catalyst amount. The highest yield of TRS was obtained at the mass ratio of catalyst to cellulose being 0.2. However, after the peak, the yield of TRS reduced. It should be that the redundant acid sites with the increasing of the catalyst amount boosted the transformation of the RS to other byproducts. Moreover, the agglomeration of the particles also caused the reduction of catalytic activity in a high catalyst amount. Figure 4d indicates the effect of the amount of water on the TRS yield. When the amount of water was increased to 7 mL, the concentration of hydrogen protons originated from water increased and facilitated the hydrolysis reaction. As the amount of water was further increased to 9 mL, the concentration of hydrogen protons was too high in a short period and led to the conversion of the produced RS to other byproducts or coke. 

#### 2.2.3. Reuse of Mt-IL in Cellulose Hydrolysis

**Figure 5:**
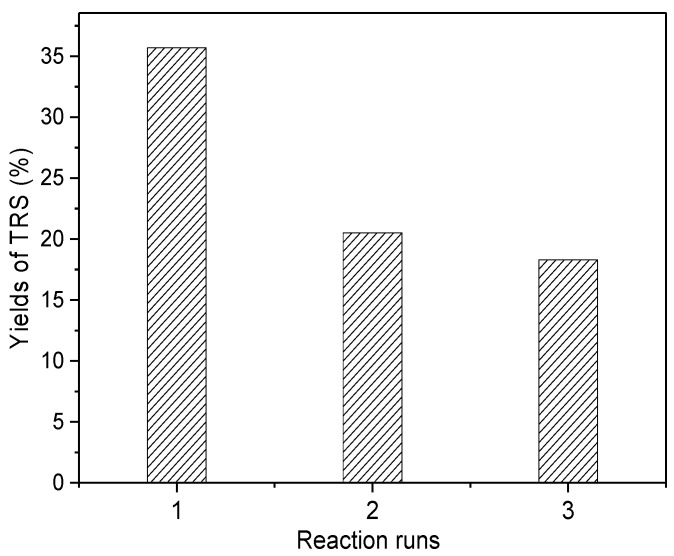
Reuse of Mt-IL catalyst for the cellulose hydrolysis.

**Figure 6:**
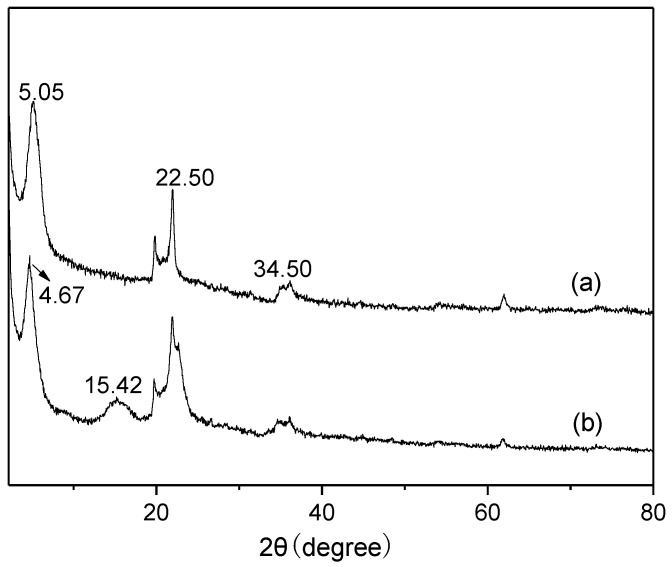
XRD patterns of fresh Mt-IL (**a**) and recycled Mt-IL after the cellulose hydrolysis (**b**).

**Figure 7:**
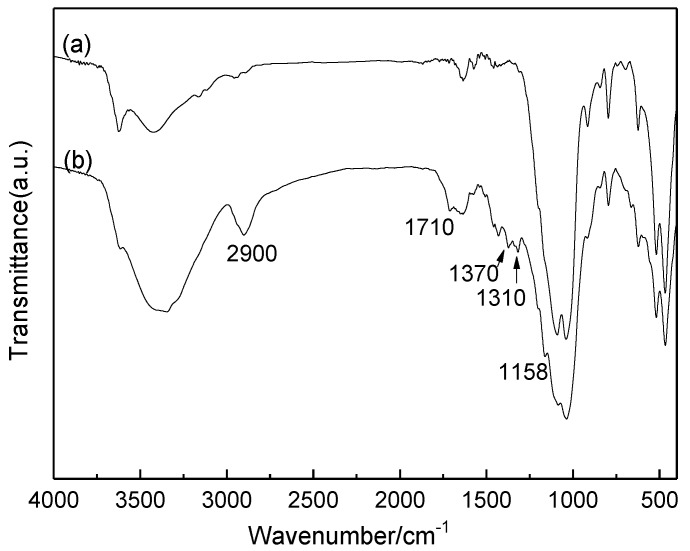
FT-IR spectra of fresh Mt-IL (**a**) and recycled Mt-IL after the cellulose hydrolysis (**b**).

Finally, the reuse of Mt-IL catalyst for the hydrolysis of cellulose in water was investigated under the optimum conditions and the results are shown in Figure 5. As shown in Figure 5, the yield of TRS decreased gradually with recycling times, but the decrease after the first run was not very serious compared to the fresh catalyst. In order to explore the deactivation, the structure of the used catalyst was characterized by XRD and FT-IR. The characterization results are shown in Figure 6 and Figure 7. According to Figure 6, the XRD pattern of the used Mt-IL showed the typical diffraction of cellulose with the reflections at 2*θ* = 15.42° for the (101) plane, 2*θ* = 22.50° for the (002) plane, and 2*θ* = 34.50° for the (040) plane [60]. Moreover, the (001) diffraction peak of Mt was further shifted from 5.05° to 4.67°, presenting that the interlayer space increased. This indicated that there existed cellulose on the used Mt-IL and the interlayer of Mt was intercalated by cellulose or the products. From Figure 7, the used Mt-IL also showed the representative bands of cellulose. The absorptions at about 2900 and 1370 cm^−1^ are related to the stretching and bending vibration of C-H. The band at 1310 cm^−1^ is assigned to the symmetric bending vibration of CH_2_. The absorption band at 1158 cm^−1^ is ascribed to the C-O-C stretching vibration at the β-(1,4)-glycosidic linkages. Furthermore, the new band at 1710 cm^−1^ was assigned to vibration of the C=O bond [61,62]. Thus, the FT-IR results also proved that cellulose existed on the surface of Mt and there were also some carbonyl compounds formed during the hydrolysis. Relating to the reuse results, it could be deduced that the deposition of cellulose and byproducts might be responsible for the deactivation of Mt-IL, and after the deposition saturation, the TRS yield changed little on the recycled catalyst.

## 3. Materials and Methods 

### 3.1. Materials

The microcrystalline cellulose powder was purchased from the ShengDeLi Synthetic Leather Material Co., Ltd., Huzhou, China, and obtained from cotton. The cellulose content was above 99 wt %. No physical or chemical pretreatments had been used to increase the non-crystalline cellulose fraction. The Ca-montmorillonite (Ca-Mt) was provided by Zhejiang ChangAn Renheng Science and Technology Co., Ltd., Hangzhou, China. The cation exchange capacity (CEC) of Mt was 80 mmol/100 g. Also, 1-methyl-1*H*-imidazol, mercaptopropyl trimethoxysilane (MPTMS), and 3-chloropropyl trimethoxy silane were purchased from Aladdin Chemicals Co., Ltd., Shanghai, China. All other chemicals (analytic purity) were commercially available products and were used without further purification.

### 3.2. Catalyst Preparation 

#### 3.2.1. Preparation of Acid-Activated Mt

The purified Ca-Mt (12 g), was dispersed in 120 mL 0.5 wt % H_2_SO_4_ and refluxed at 80 °C for 1.5 h. Then the slurry was cooled, filtered, and washed thoroughly with distilled water three times. The product was dried at 80 °C for 12 h. The activated Mt was designated as HMt.

#### 3.2.2. Preparation of Mt-SO_3_H

In a typical procedure, 12 g Ca-Mt in 360 mL water was stirred for 30 min at room temperature, and then 4 mL MPTMS was added into it. Finally, the mixture was stirred at 80 °C for 2.5 h to obtain thiol functionalized Mt (Mt-SH). Then the slurry was cooled, filtered, and washed thoroughly with anhydrous ethanol two times, and the thiol group was oxidized to sulfonic acid using H_2_O_2_ in the presence of ethanol at 60 °C for 4 h (58 mL of 30% H_2_O_2_ in 174 mL ethanol for 12 g of Mt-SH). After that, the slurry was cooled, filtered, and washed thoroughly with distilled water three times. The product was dried at 80 °C for 12 h. The amount of -SO_3_H was adjusted by the concentration of MPTMS and the synthesized samples were designated as Mt-SO_3_H-1, Mt-SO_3_H-2, and Mt-SO_3_H-3, respectively.

#### 3.2.3. Preparation of Mt-IL

Firstly, 1-(trimethoxy propyl silane)-3-methyl imidazolium chloride (Si(MeO)_3_PMIMCl) was synthesized. Four milliliters of 1-methyl-1*H*-imidazol and 10 mL of 3-chloropropyl trimethoxy silane were mixed in 100 mL of toluene and the mixture stirred at the reflux temperature of toluene (120 °C) for 12 h. Finally, the toluene was removed by rotary evaporation and an orange viscous liquid Si(MeO)_3_PMIMCl was obtained. 

Then, the Ca-Mt was mixed with Si(MeO)_3_PMIMCl in anhydrous ethanol (100 mL) at 90 °C for 12 h. After silanization, the precipitate was filtered and washed with distilled water three times. The product was dried at 80 °C for 12 h. The sample was designated as Mt-IL. 

### 3.3. Catalytic Conversion of Cellulose

The hydrolysis reaction was carried out in a Teflon-lined stainless steel autoclave (25 mL). A certain amount of distilled water, cellulose, and catalyst were introduced into the autoclave. The reaction was carried out between 170 and 220 °C under antogenetic pressures. After the reaction, the catalyst and the unreacted cellulose were removed by filtration. The liquid products were analyzed at 540 nm using 3,5-Dinitrosalicylic acid method by visible spectrophotometer manufactured by Shanghai Precision & Science Instruments Co. Ltd. Cellulose conversions were determined by the change of cellulose weight before and after the reaction, with an uncertainty of ±3%. The yield of reducing sugar was calculated from the equation: yield (%) = (weight of reducing sugar in the products)/(weight of cellulose put into the reactor) × 100%. The concentration of 5-HMF was analyzed by a Waters 2695 series HPLC equipped with an ultraviolet detector applying an InertSustain C18 (4.6 mm × 250 mm × 5 μm), and the yield of 5-HMF was calculated based on the calibration curves.

### 3.4. Catalyst Characterization

The X-ray diffraction (XRD) measurements were collected using a PANAlytical X’Pert PRO diffractometer between 2° and 80° (2θ) with a scanning rate of 0.1 °/s, employing Cu Kα radiation (λ = 1.54056 Å). Fourier transform infrared (FT-IR) spectra were recorded between 4000 and 400 cm^−1^ using a Nicolet 6700 Fourier transform spectrometer. The samples were dried at 110 °C, mixed with KBr, and exposed to infrared light. The pellets were immediately measured after preparation under ambient conditions in the mid-infrared region. The spectra were the result of averaging 32 scans at wavelengths ranging from 4000 to 400 cm^−1^. Thermal analysis of nanocomposites was carried out using thermogravimetric-differential thermogravimetric (TG-DTG) methods on a Mettler Toledo thermobalance. TG/DTG curves were recorded with a 10 °C/min heating rate under air atmosphere between 30 and 800 °C.

The measured process for acidic sites on the catalysts was described as follows: a catalyst (0.05 g) was treated with 0.01 mol/L of NaCl solution (20 mL) for 1 h at 20–50 °C under ultrasonic vibration. After centrifugal separation, the supernatant solution was titrated by 0.01 mol/L of NaOH solution using phenolphthalein as an indicative.

## 4. Conclusions

In summary, an efficient and friendly catalyst was prepared for the hydrolysis of cellulose to reducing sugars in water. The montmorillonite grafted by non-acidic ionic liquid (Mt-IL) catalyst was active for the hydrolysis of cellulose. The highest TRS yield of 35.7% was obtained at 200 °C with the mass ratio of catalyst to cellulose of 0.2 for 120 min on the Mt-IL catalyst. The high TRS yield for Mt-IL should be attributed to the synergistic effect of the dissolution of cellulose by IL and the exposed metal ions on the layer with water. Although the yield of TRS on Mt-IL decreased gradually with recycling runs, its decrease was not very serious after the first run. The deposition of cellulose and byproducts might be responsible for the deactivation of Mt-IL. This work provides a promising strategy for the effective hydrolysis of cellulose into reducing sugars as well as into other chemicals in water with non-acidic IL.

## Figures and Tables

**Table 1:** Hydrolysis of cellulose catalyzed by Mt and functional Mt in water **^a.^**

Entry	Catalysts	Content of Acidic Sites (mmol·g^−1^)	TRS Yield (%)	5-HMF Yield (%)
1	Mt	0.012	7.9	0.4
2	HMt	0.320	14.4	1.0
3	Mt-SO_3_H-1	0.236	19.3	1.6
4	Mt-SO_3_H-2	0.296	24.5	1.7
5	Mt-SO_3_H-3	0.532	24.6	1.8
6	Mt-IL	0.056	35.7	1.9

^a^ Catalyst: 0.1 g, cellulose: 0.5 g; water: 7.0 mL, reaction temperature and time: 200 °C and 120 min. TRS: total reducing sugars.
